# Anti-tumor activities of probiotics in cervical cancer

**DOI:** 10.1186/s13048-020-00668-x

**Published:** 2020-06-11

**Authors:** Moghaddaseh Jahanshahi, Parisa Maleki Dana, Bita Badehnoosh, Zatollah Asemi, Jamal Hallajzadeh, Mohammad Ali Mansournia, Bahman Yousefi, Bahram Moazzami, Shahla Chaichian

**Affiliations:** 1 Clinical Research Development Center (CRDC), Sayad Shirazi Hospital, Golestan University of Medical Sciences, Gorgan, Iran; 2 Research Center for Biochemistry and Nutrition in Metabolic Diseases, Institute for Basic Sciences, Kashan University of Medical Sciences, Kashan, Iran; 3 Department of Gynecology and Obstetrics, School of Medicine, Alborz University of Medical Sciences, Karaj, Iran; 4 Department of Biochemistry and Nutrition, Research Center for Evidence-Based Health Management, Maragheh University of Medical Sciences, Maragheh, Iran; 5 Department of Epidemiology and Biostatistics, School of Public Health, Tehran University of Medical Sciences, Tehran, Iran; 6 Stem Cell Research Center, Tabriz University of Medical Sciences, Tabriz, Iran; 7 Department of Biochemistry, Faculty of Medicine, Tabriz University of Medical Sciences, Tabriz, Iran; 8 Pars Advanced and Minimally Invasive Medical Manners Research Center, Pars Hospital, Iran University of Medical Sciences, Tehran, Iran

**Keywords:** Probiotics, Cervical cancer, Lactobacillus, Radiotherapy-induced diarrhea, Apoptosis

## Abstract

Cervical cancer is considered as an important malignancy among women worldwide. Currently-used treatments of cervical cancer are reported to be cytotoxic for patients. Moreover, these therapies have shown some side effects which can negatively affect the lives of women suffering from this cancer. Therefore, there is need for anti-tumor agents that are less toxic than common therapeutic drugs. Besides, applying agents for preventing or reducing the side effects of cervical cancer therapies can be effective in improving the life quality of cervical cancer patients. Studies have shown that probiotics have several effects on biological processes. One of the most prominent aspects in which probiotics play a role is in the field of cancer. There are multiple studies which have focused on the functions of probiotics in diagnosis, prevention, or treatment of cancer. Besides their direct anti-tumor activities, probiotics can be used as an additional agent for enhancing or modulating other diagnostic and therapeutic methods. Herein, the effects of probiotics on cervical cancer cells are discussed, which may be useful in the prevention and treatment of this cancer. We review the studies concerned with the roles of probiotics in modulating and reducing the gastrointestinal adverse effects caused by cervical cancer therapies. Furthermore, we cover the investigations focusing on the combination of probiotics with other drugs for diagnosis or treatment of cervical cancer.

## Background

There are thousands of bacterial species in the human body, which provide and maintain the health of their host [1, 2]. Nine percentage of microbial species are located in the urogenital tract. Thus, there should be a balancing connection between the immune system of host and microbiota that exist in urogenital tract [1, 3, 4]. Disruptions in this balance may lead to cervical cancer by allowing infectious agents to grow in this area. Currently-used chemotherapeutic drugs can result in cytotoxic effects in patients suffering from cervical cancer. Besides, there are side effects caused by therapies which are unavoidable [5]. Therefore, there is need for anti-tumor agents that are non-toxic or cause less toxicity than other therapeutic agents. Also, applying drugs for preventing or reducing the side effects of cervical cancer treatment would be effective in enhancing the life quality of cervical cancer patients [6].

Herein, the effects of probiotics on cervical cancer cells which may be useful in the prevention and/or treatment of this cancer are discussed. We review the studies concerned with the roles of probiotics in modulating and reducing the gastrointestinal adverse effects caused by cervical cancer therapies. Furthermore, we cover the investigations focusing on the combination of probiotics with other drugs for diagnosis or treatment of cervical cancer.

## Cervical cancer

### Epidemiology

Cervical cancer is considered a global health concern since it is the fourth common malignancy among women worldwide [7]. Cervical cancer comprises 4% of all cancers that are diagnosed all around the world [8]. Based on morphological characteristics, there are three main subtypes of cervical cancer, including squamous cell carcinoma, adenocarcinoma, and adenosquamous carcinoma. All of these subtypes have the same one-year net survival rate of 85% [9]. Adenocarcinoma and squamous cell carcinoma, the two most frequent subtypes, are responsible for nearly 25 and 70% of cervical cancer, respectively [10, 11]. In addition, there are metabolic disturbances in women with polycystic ovarian syndrome [12, 13], which may increase the risk of cervical cancer. Noteworthy, incidence rates and deaths of cervical cancer have been progressively reduced in the well-developed countries because of HPV vaccination and cancer screening programs [14, 15].

### Risk factors

One of the well-studied risk factors of cervical cancer is HPV chronic infection. Besides, there are other factors implied to raise the risk of this cancer including smoking, co-infection with type 2 herpes simplex, co-infection with human immunodeficiency virus, high parity, and consumption of oral contraceptives for a long time [16, 17]. Some risk factors of cervical cancer are related to acquiring HPV infection or inefficient response of the immune system to HPV infection such as sexual debut at early ages, high-risk sexual partners, multiple sexual partners, immunosuppression, history of sexually transmitted infection (STI), and history of HPV-related vulvar or vaginal dysplasia [11].

### Prevention

HPV vaccination and screening of cervical cancer can be applied to prevent this disease. HPV, which is the most common STI globally, accounts for virtually all (99.7%) of the cervical cancer cases [18]. More than two-thirds of cervical cancers and precancerous lesions are caused by two HPV types that are type 16 and 18 [19, 20]. There are two approved vaccines for HPV, including bivalent vaccine and quadrivalent vaccine. The former one is against 16 and 18 types of HPV types and quadrivalent vaccine (against HPV types 6, 11, 16, and 18) [21]. Predictions show that the incidence of cervical cancer will decrease up to 83% by a 90% coverage of vaccine [22]. The pre-cancerous condition can be detected and treated by a secondary prevention method called cervical cancer screening, which is helpful in malignancy prevention [23]. In high-resource countries, early diagnosis and treatment of precancerous lesions can prevent up to 80% of cervical cancer [24]. Cytology is the most frequent tool for cervical cancer screening which is used in developed countries [23]. In patients whose screening has shown premalignant cervical lesions such as cervical intraepithelial neoplasia, colposcopy is done to confirm the finding [25]. In low-resource countries, a feasible see-and treat method is visual inspection with acetic acid (VIA) or visual inspection with Lugol’s iodine (VILI) [26]. However, coverage of screening programs is still low despite the increase in the availability of them [23].

### Clinical presentation and diagnosis

Cervical cancer does not usually show any symptoms in its early stages [27]. Post-coital vaginal bleeding, abnormal vaginal bleeding, and malodorous vaginal discharge are symptoms of this cancer [28, 29]. Passages of urine and feces through the vagina are symptoms for an invasion of the bladder and rectum, respectively [30, 31]. A pelvic examination and cervical cytology, as well as visualization of the cervix and vaginal mucosa by a speculum, are needed when a woman presented with cervical cancer symptoms [11]. However, the cervix may look normal in observations when the cancer is micro-invasive or occurred in the endocervical canal [11]. There are possibly palpable swollen and hardened lymph nodes in inguinal and supraclavicular regions in patients with the advanced-stage disease [11]. In distant metastasis, positron emission tomography (PET)-computed tomography and fluoro-2-deoxy-D-glucose PET (FDG-PET) are two efficient methods for diagnosis [32].

### Treatment

Currently, cervical cancer management is dependent on a multidisciplinary team approach [33]. In the early stages of the disease, there are multiple treating approaches that can be used, including surgery, radiation, neoadjuvant chemotherapy, and fertility preservation surgery [33]. The standard treatment for patients suffering from locally advanced cervical cancer is concurrent chemoradiation (CCRT) in which cisplatin is used alone or in combination with other drugs [33]. In nearly 30–40% of cases, there is a lack of complete response [33]. Bevacizumab is an inhibitor of vascular endothelial growth factor [34]. In the recurrent or metastatic form of the disease, bevacizumab administration along with chemotherapy has linked to enhanced survival [33]. Different treating options are available for managing metastatic patients who suffer from lung metastasis, bone metastasis, solitary brain metastasis, or multiple brain metastases. These treating approaches include chemotherapy and surgery, chemotherapy and bone irradiation, craniotomy or stereotactic radiosurgery combined with radiotherapy, and chemotherapy and palliative brain radiation, respectively [32]. Moreover, CCRT followed by chemotherapy are helpful in lymph metastatic patients [32]. Investigations have provided promising findings of novel therapeutic methods such as inhibitors of the immune checkpoint, inhibitors of tyrosine kinase, and targeted therapy with anti-angiogenesis drugs [33].

## Probiotics

### What are probiotics?

Probiotic is a Latin-derived word with the meaning of “for life” [35]. Fermented products which include cheese, bread, wine, beer, and kefir were widely used for their nutritional and therapeutic benefits long before the identification of probiotics [35]. Living microorganisms that provide several beneficial impacts to their host in case of proper amounts are called probiotics [36, 37]. This broad definition comprises drugs containing probiotics, conventional foods, medical foods, dietary supplements, infant formula, animal feed, non-oral probiotics, and defined microbial consortia [38]. The focus of earlier studies were nutritional roles of probiotics which have been used as a food form or a dietary supplement [39]. However, recent probiotics research has concentrated on its functions in the medical and therapeutic fields [40]. The natural microbiota of intestine comprises the majority of probiotics, which are particularly lactic acid bacteria, including *Enterococcus, Vagococcus, Weisella, Leuconostoc, Lactococcus, Streptococcus, Tetragenococcus, Bifidobacterium, Carnobacterium, Oenococcus, Pedicoccus, Lactobacillus, Escherichia coli*, and *Saccharomyces* [41, 42]*.* Noteworthy, not all bacteria of the same species have the same characteristics and may have different effects on the organism. Besides, all strains are not probiotic [43].

### Applications of probiotics

Probiotics applications in the prevention or treatment of various diseases have been studied in several investigations. Probiotics are involved in various processes of the digestive system such as digestion, metabolism, innate immunity of epithelial cells, eliminating pathogens, and communication between brain and gut through their adhesion to human intestines [44, 45]. Evidence has shown the beneficial effects of gut microorganisms producing nontoxic metabolites in different nutritional and clinical aspects [46–48]. Probiotics and fermented non-digestible food products, prebiotics, work together and have been shown to have several beneficial characteristics such as anti-pathogenic, anti-inflammation, antidiabetic, and anti-obesity [49, 50]. Probiotics are also involved in immune processes such as increasing antibody responses and inhibiting the mononuclear cells proliferation [51, 52]. Probiotics have been used for a variety of gastrointestinal problems including irritable bowel syndrome, constipation, ulcerative colitis, and necrotizing enterocolitis [53–57]. Increasing anti-inflammatory cytokines such as interleukin-10 (IL-10) and IL-12 and reducing pro-inflammatory cytokines such as IL-1β and IL-6 are anti-inflammatory activities of probiotics which have been investigated in different diseases [58, 59]. Studies indicated that probiotics can be helpful in autoimmune diseases including rheumatoid arthritis, systemic lupus erythematosus, and multiple sclerosis [58, 60–62]. Other findings have also revealed that probiotics are beneficial for high cholesterol levels in serum, allergy, vulvovaginal candidiasis, HIV, and cancer [63–66].

### Safety and side effects of probiotics

In general, probiotics are safe [67]. Although, in patients who are immunocompromised or when someone is considered severely ill, precautions should be observed [67]. Rash, hiccups, nausea, constipation, and flatulence are some of the most common adverse effects of probiotics [67]. There are other probiotics side effects such as systemic infections, damaging metabolic activities, and transferring harmful genes such as resistance to antimicrobial agents [68]. In some rare cases, using Lactobacillus have led to the liver abscess, sepsis, and endocarditis [67]. There is evidence indicating that Bacillus subtilis can cause cholangitis, bacteremia, and sepsis [69]. Also, it is reported that S. boulardii may lead to fungal sepsis [70]. Altogether, probiotic-related risk of infection is the same as infection risk of commensal bacterial strains, and it seems that the advantages of probiotics outweigh its risks [71].

### Probiotics and cancer

One of the most prominent aspects in which probiotics play a role is in the field of cancer. Probiotics act as a double-edged sword in this field. Accumulative investigations have revealed that microbiota can participate in the process of carcinogenesis of many types of cancers, including gynecological cancers. For instance in cervical cancer, dysbiosis is approved to have influences on both HPV infection by affecting HPV acquisition, clearance, and persistence and host immune response by affecting the levels of immune system proteins such as TGFβ1 [72]. Various studies have focused on the functions of probiotics in diagnosis, prevention, or treatment of cancer. Besides their direct involvement in the mentioned areas of cancers, probiotics can be used as an additional agent in enhancing or modulating other diagnostic and therapeutic methods. Kailasapathy et al. [73] have explained some underlying mechanisms by which probiotics may play their role as antitumor agents, such as activating the immune system of the host, changing transit time and motility of the colon, suppressing pro-carcinogens and carcinogens, inhibiting bacteria that are involved in the transformation of pro-carcinogens to carcinogens, and reducing intestinal pH [73]. Findings demonstrate that probiotics make an impact on biological processes which are involved in cancer, including apoptosis, oxidative stress, proliferation, inflammation, and metastasis [74–77]. Long-time exposure to aflatoxins, especially aflatoxin B_1_ has been related to a higher risk of liver cancer development [78]. It is suggested that probiotic supplements may be useful in preventing liver cancer due to their ability to reduce the aflatoxin exposure dose that is biologically effective [79]. Zonulin protein is involved in regulating intestinal permeability [80]. In colorectal cancer patients who undergo colectomy, findings indicate that administering probiotics perioperative results in a reduction in postoperative septicemia as well as zonulin concentrations in serum [80]. In breast cancer, long-term administration of probiotic strain *Lactobacillus plantarum* LS/07 has shown immunomodulatory effects such as reducing tumor necrosis factor (TNF)-α and increasing Cd4(+) T-cells [81]. One of the common side effects of systemic chemotherapy is diarrhea [82]. It has been revealed that probiotic Clostridium butyricum can reduce chemotherapy-induced diarrhea and systemic inflammatory responses in lung cancer patients [83]. Altogether, the role of probiotics in cancer is extensive and we will provide a more detailed description of their functions in cervical cancer.

## Probiotics and cervical cancer

It was reported that probiotics can be efficient in different gynecological diseases [84]. Herein, we look into studies investigating how probiotics can be useful in the prevention, diagnosis, and treatment of cervical cancer.

### Roles of probiotics on cervical cancer cells

Wang et al. [85] have shown that Lactobacillus supernatants (LS), *L. crispatus*, L. jensenii, and L. gasseri, inhibit the proliferation of Caski cells and cause some morphological alterations. Through incubating cells with LS, the number of S phase cells increased significantly; meanwhile, G2/M phase cells decreased [85]. E6 and E7 are two genes from eight genes that are encoded by HPV [86]. These two genes encode the proteins that are linked with p53 and pRB tumor suppressors and are required for conversion to malignancy [86]. LS treatment results in a reduction in the expression of CDK2, cyclin A, and HPV oncogenes (E6 and E7) [85]. Moreover, p21 expression was enhanced in LS-treated cells [85]. *Lactobacillus plantarum* bacteria, which were isolated from vaginal secretions of young adult and adolescent women, are observed to have probiotic features and anticancer activities against HeLa cervical cancer line [87]. Another study on HeLa cell line revealed that human milk-isolated Lactobacillus strains (Lactobacillus casei SR1, Lactobacillus casei SR2, and *Lactobacillus paracasei* SR4) have remarkable probiotic activities, including antibiotic susceptibility, antioxidant roles, low pH resistance, and resistance to high levels of bile salts [88]. Furthermore, findings indicate that cell-free culture supernatants have anticancer activities, such as downregulating BCL-2 and upregulating apoptotic genes (caspase3, caspase8, caspase9, BAX, and BAD) [88]. Sungur et al. [89] reported that human vagina-isolated L. gasseri strains, G10 and H15, inhibits the HeLa cells proliferation. Lactobacillus exopolysaccharides exert cytotoxic effects on cancer cells and decrease their proliferation [90]. While lyophilized exopolysaccharides of L. gasseri strains led to apoptosis in HeLa cells, G10 apoptosis induction has related to Bax and Caspase3 upregulation [89]. Besides, strains of L.gasseri reduce TNF-α and increase IL-10, which leads to their anti-inflammatory effect on cervical cancer [89]. It is observed that treating HeLa cells with supernatants of *Lactobacillus rhamnosus* and lactobacillus crispatus reduces the expression of CASP3 gene as well as MMP2 and MMP9, which causes an inhibitory effect on metastasis [91]. Findings show that Bifidobacterium adolescentis SPM1005-A exerts an antiviral activity in SiHa cervical cell line, which express HPV type 16 and may prevent this cancer [92]. Treating cells with this bacterium has been shown to reduce the E6 and E7 oncogenes at mRNA and protein levels of E6 [92]. L. gasseri 3396 and L. cripatus 2743 are two probiotic strains [93]. It is observed that L. cripatus 2743 has an inhibitory effect on the expression of E6 and E7 at the mRNA level [93]. Meanwhile, L. gasseri 3396 has a smaller inhibitory impact on the E6 gene without any significant effect on the E7 gene [93] (Table 1).

**Table 1:** Studies investigating probiotics effects on cervical cancer cells

Probiotic	Cell line	Findings	References
supernatants of *L. crispatus*, L. jensenii, and L. gasseri	Caski	Inhibition of the viability by regulation of HPV oncogenes and cell cycle-related genes	[85]
Vagina-isolated *L. plantarum*	HeLa	Suppression of proliferation and induction of apoptosis	[87]
Milk-isolated L. casei and *L. paracasei*	HeLa	Induction of apoptosis	[88]
Vagina-isolated L. gasseri	HeLa	Inflammation and proliferation were reduced and apoptosis was increased	[89]
*Supernatants of* *L. rhamnosus* and *L. crispatus*	HeLa	Proliferation and metastasis were suppressed	[91]
*Bifidobacterium adolescentis* SPM1005-A	SiHa	Suppression of E6 and E7 oncogenes	[92]

### Probiotics effects on gastrointestinal problems of cervical cancer patients

As we mentioned earlier, radiotherapy is an effective method for treating cervical cancer patients [94]. However, radiotherapy has multiple side effects causing an extra burden to patients such as nausea, vomiting, and diarrhea [95, 96]. Radiotherapy-induced diarrhea is one the most frequently occurred side effects, which can involve up to 80% of patients and may lead to severe complications [97, 98]. It is suggested that this side effect is the result of lactose and bile acids malabsorption [45]. Moreover, it may be due to the alterations in the intestinal flora and motility [45]. These events cause an impairment in immune roles of the gastrointestinal tract as well as its secretion and absorption [45].

There are some investigations concerned with the roles of probiotics in preventing or modulating the gastrointestinal problems which cervical cancer patients face due to cancer itself or the treatments. It is reported that administering a probiotic which contains live Lactobacillus acidophilus LA-5 plus Bifidobacterium animalis subsp. lactis BB-12 leads to reduced incidence of mild to moderate and severe diarrhea in cervical cancer patients who undergo beam pelvic radiotherapy [99]. In a study, a probiotic drink has been used for patients with cervical carcinoma who received radiotherapy and weekly cisplatin. Results showed that giving Lactobacillus casei DN-114001 as a probiotic has a significant effect on stool consistency. However, there was no reduction in the incidence of diarrhea in patients who received probiotic drink [100]. Another trial reported that fewer patients who were assigned to VSL#3 had radiation-induced diarrhea compared to the placebo group. A higher percentage of patients in the placebo group experienced grade 3 or 4 diarrhea compared to VSL#3 patients. Also, daily bowel movements were lower in patients who receive VSL#3 [101]. Therefore, probiotics can be considered as safe and effective agents for reducing radiotherapy-induced diarrhea in patients with cervical cancer. Symbiotics are products consisting of prebiotics and probiotics that include Lactobacilli, Bacteroides, Bifidobasteria, and Eubacterium. This product plays several beneficial roles including inactivating carcinogens, maintaining the integrity of intestine, immunomodulation, and anti-inflammatory activities [102, 103]. Findings demonstrated that patients who were given symbiotic showed lower levels of fecal calprotectin as well as less frequent and less intense vomiting [104]. Thus, symbiotic supplementation may be also helpful in reducing gastrointestinal adverse effects of cervical cancer patients (Table 2).

**Table 2:** Studies concerned with the roles of probiotics in preventing or reducing the adverse effects of cervical cancer therapies on gastrointestinal tract

Type of probiotics	Dosage	Duration of probiotic consumption	Results	References
L. acidophilus LA-5 plus Bifidobacterium animalissubsp. Lactis BB-12	One capsule containing 1.75 billion lyophilized live bacteria t.i.d.	From the first day until the end of radiotherapy	Severity and incidence of radiotherapy-induced diarrhea were reduced	[99]
L. acidophilus plus bifidobacterium bifidum	2 × 10^9^ units of a L. acidophilus plus bifidobacterium bifidum b.i.d.	Beginning 7 days before starting radiotherapy and continuing every day during radiotherapy	Stool consistency was improved and need for anti-diarrheal medication was reduced	[105]
L. casei DN-114001	96 mL of a liquid yogurt containing approximately 108 CFU/g of L. casei DN-114001 t.i.d.	Beginning 7 days before starting radiotherapy and continuing every day during radiotherapy	Stool consistency was improved but there was no reduction in the incidence of radiotherapy-induced diarrhea	[100]
VSL#3	one sachet containing 450 billion/g of bacteria, including four strains of lactobacilli, three strains of bifidobacteria, and one strain of *Streptococcus salivarius* t.i.d.	From the first day of radiation therapy until the end of the scheduled cycles of radiotherapy	Daily bowel movements and incidence of radiotherapy-induced diarrhea were reduced	[101]
Sybmbotic	symbiotic containing 1 × 107 (CFU)/g biogel of L. acidophilus NCFM, Bifidobacterium lactis Bi-07 1 × 106 CFU/g biogel, and blue agave inulin t.i.d.	Seven weeks	Fecal calprotectin and frequency and intensity of vomiting were reduced	[104]

Colony-forming unit, CFU; b.i.d., twice a day; t.i.d., three times a day

### Probiotics provide a better diagnosis of cervical cancer

Bacteria can be used as a diagnostic device through engineering techniques that make the bacteria sense a specific molecule in the human body and consequently produce a signal [106]. For instance, Danino et al. [107] developed a probiotic-based diagnostic that is orally administered. They showed that using *Escherichia coli* Nissle 1917 is a non-invasive approach for producing signals that are detectable in urine; leading to the identification of liver metastasis. Danino et al. [107] showed that by programming, probiotics can be used for delivering gene circuits to unhealthy tissue microenvironment. L. lactis has been also used for detecting a molecule that is produced by Vibrio cholera [108]. Through developing a diagnostic circuit in this bacteria, the color of the host’s feces changes when there is an infection, which provides an early alert of cholera [108]. Thus, engineering may make it possible to use probiotics as a diagnostic device in cervical cancer. *Lactobacillus rhamnosus* GR-1 and *Lactobacillus reuteri* RC-14 are two probiotic strains which have shown desirable effects on gynecological diseases [109, 110]. Evidence suggests that using *Lactobacillus rhamnosus* GR-1 and *Lactobacillus reuteri* RC-14 along with anti-infective agents leads to a reduction in false negative and false positive results of cervical malignancies; thus, cytological diagnoses are more reliable with this method [111]. Compared to when using anti-infective drugs alone, applying these probiotic strains provide a higher percentage of vaginal microflora normalization in patients who have vaginal infection [111]. A study investigated the impacts of probiotic strains on the quality of cervical smear and clearance of genital high-risk human papilloma virus [112]. It is observed that *Lactobacillus rhamnosus* GR-1 and *Lactobacillus reuteri* RC-14 significantly reduced the rates of unsatisfactory and mildly abnormal cervical smears. However, they did not affect the clearance of HPV [112].

### Combination of probiotics with other cervical cancer therapies

Cisplatin has been one of the most important chemotherapeutics in patients with advanced cervical cancer [113]. It is reported that cisplatin pro-apoptotic and antigrowth effects are enhanced by co-treatment with Lactobacillus bacteria in mouse models with lung cancer [114]. Co-treatment with Lactobacillus led to the upregulation of IFN-γ, PRF1, and GZMB; resulting in a better anti-tumor response to cisplatin [114]. PD-L1 blockade has shown a better anti-tumor activity in mice harbouring unfavorable gut microbiota by orally administrating Bifidobacterium probiotic. Also, it is observed that this combined treatment prevented tumor growth [115]. A study of 228 patients with cervical cancer at stage IIIB demonstrated that combining LC9018, a response modifier that is derived from heat-killed Lactobacillus casei YIT9018, with radiation therapy would improve the outcomes [116]. Okawa et al. concluded that patients who received LC9018 as an adjuvant agent had a longer survival and relapse-free interval than patients who received radiation therapy alone. Moreover, these patients showed less severe radiation-induced leukopenia [116]. In a recent study, Negi et al. [117] provided a drug delivery approach in which probiotic strains were applied. They concluded that the pessaries which contain cisplatin and probiotic biomass can be a better therapeutic method for cervical cancer since probiotic strains showed favorable effects such as scavenging free radicals [117]. In contrast, Kim et al. [118] reported that the extract of L. casei showed no synergistic effects with anti-cancer drugs to suppress the Hela and Caski cancer cells growth. Although there are many studies on the role of probiotics in cervical cancer, little is known about their synergistic effect with other drugs of this cancer. However, probiotics may be potential agents that can increase the antitumor effects of other drugs [119].

### Effects of probiotics on the therapeutic targets of cervical cancer

While the majority of articles focus on the direct role of probiotics on cervical cancer cells or their role in reducing the side effects of other treatments, evidence from other cancer types suggests that probiotics may affect other drug targets of this cancer. However, studies in this area are limited and more research is needed.

It is reported that miR-29a and miR-21 are the most common downregulated and upregulated miRNAs, respectively, involved in the progression of invasive cervical cancer [120]. There some other dysregulated miRNAs in cervical cancer, including miR-10a, miR-20b, miR-106, miR-16 and miR-9 [120]. Moreover, exfoliated cells of the cervix that are associated with cancer progression present dysregulated miR-375, miR-125, and miR-34a [120]. It is found that vaginal-isolated Lactobacillus lactis leads to the downregulation of TLR-4, miR-200b, and miR-21, which is associated with apoptosis induction [121]. Also, it is observed that the probiotic *Escherichia coli* Nissle 1917 restores the expression levels of some miRNAs, including miR-143, miR-155, and miR-375 [122]. MiR-21 is reported to have a significantly higher level in the human umbilical vein endothelial cell that were cultured in different conditions of Lactobacillus acidophilus such as water extract, culture media, culture-filtered and unfiltered supernatants. Furthermore, cells treated with L. acidophilus culture media have shown the highest miR-21 level compared to cells treated with other conditions of L. acidophilus Conversely, *Escherichia coli* lipopolysaccharide resulted in a decrease in miR-21 levels compared to the control group [123].

HPV infection triggers the activation of NF-κB that affects both innate and adaptive immune responses. The virus eliminates the inhibitory effects of the immune system by downregulating NF-κB; resulting in a persistent status of infection [124]. Then, NF-κB reactivates during the transformation to cervical cancer. NF-κB leads to the transcriptions of genes that are involved in proliferation and metastasis as well as cell immortality and VEGF-dependent angiogenesis [124]. Probiotics are capable of inhibiting the inflammation through suppressing different signaling pathways such as the nuclear factor-κB (NF-κB) pathway. Besides, lipopolysaccharides binding to the CD14 receptor can be inhibited by probiotics; leading to a decrease in overall NF-κB activation as well as the production of pro-inflammatory cytokines [125]. Findings show that anti-inflammatory *Lactobacillus plantarum* NK3 and Bifidobacterium longum NK49 suppress the activation of NF-*κ*B and the expression of TNF-*α* in the mice vagina and uterus [126]. Lactobacillus johnsonii, Bifidobacterium longum, *Lactobacillus plantarum*, Lactobacillus delbruekii, and *Lactobacillus fermentum* are also observed to inhibit NF-κB activation [127–129].

Findings indicate that STAT3 has a potential role in the development of cervical cancer [130, 131]. HPV positive cervical cancer cells have a higher level of constitutively activate STAT3 compared to HPV-negative cells [132]. Moreover, Shukla et al. reported that STAT3 is associated with the conversion of precancerous cervical lesions to cancer [132]. It is observed that pro-inflammatory cytokine IL-6 mediate the autocrine activation of STAT3 in HPV^+^ cervical cells [133]. Phosphorylation of STAT3 increases with both conditioned media from HPV^+^ cervical cells and recombinant IL-6 [133]. Probiotic agent VSL#3 has chemopreventive roles against the colitis-associated carcinogenesis. Evidence has revealed that VSL#3 exerts its function through the suppression of IL-6/STAT3 [134]. Zhou et al. [135] also reported that exopolysaccharides from *Lactobacillus plantarum* NCU116 promote the binding of STAT3 to the promotor of occluding and ZO-1. Besides, treatment with this exopolysaccharide results in STAT3 knockdown; leading to a reduction in the expressions of occludin and ZO-1 [135]. On the other hand, it is reported that Lactobacillus enhances the generation of intestinal stem cells by activating STAT3 signaling pathway [136].

## Conclusions

**Fig. 1:**
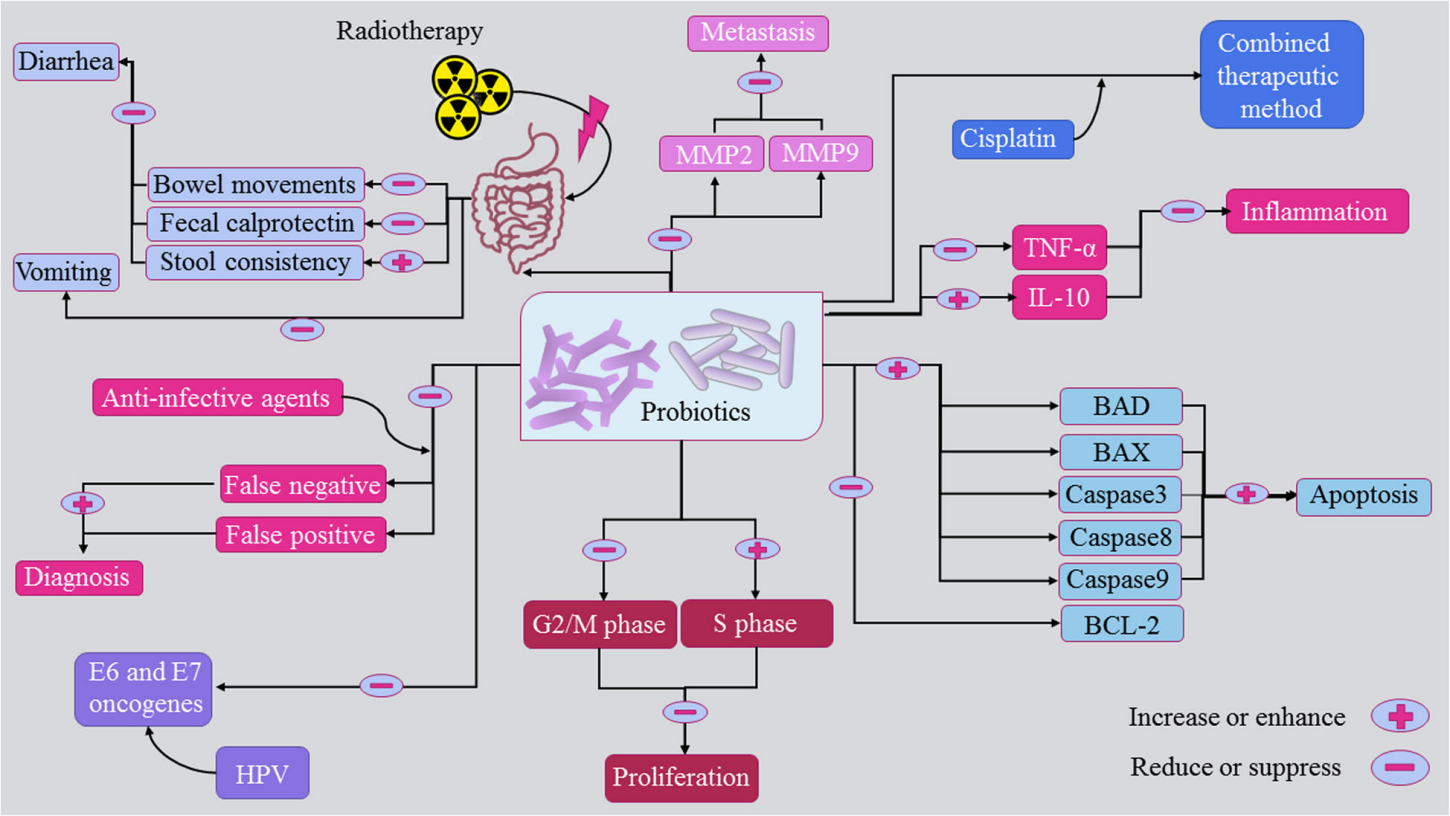
Representation of how probiotics are involved in the prevention, diagnosis, and treatment of cervical cancer

Cervical cancer is one of the most important cancers among women. Probiotics are beneficial in different cancers. Some studies have been focused on the effects of the probiotic on cervical cancer (Fig. 1). Findings have shown that probiotics have remarkable abilities which may lead to the prevention or treatment of cervical cancer including induction of apoptosis, inhibiting proliferation, reducing inflammation, and suppressing metastasis. Evidence suggests that concurrent use of probiotics with other therapeutic drugs results in an improvement in treatments. When combined with anti-infective drugs, probiotics have been shown to provide a better diagnostic method for cervical cancer patients. Besides, clinical trials are indicating that administering probiotics have a significant impact on gastrointestinal side effects which are caused by cervical cancer therapies. However, more research, especially clinical trials, should be done to understand the precise effects of these anti-tumor agents. Besides, the side effects of probiotics in cervical cancer patients should also be carefully investigated. Overall, probiotics appear to be potential anticancer agents which can be used in the prevention and treatment of cervical cancer.

## Abbreviations

HPV: Human papillomavirus
VIA: Visual inspection with acetic acid
VILI: Visual inspection with Lugol’s iodine
TNF: Tumor necrosis factor
STI: Sexually transmitted infection
PET: Positron emission tomography
LS: Lactobacillus supernatants
CCRT: Concurrent chemo-radiation
IL: Interleukin

## References

[1] Nami Y, Abdullah N, Haghshenas B, Radiah D, Rosli R, Khosroushahi AY (2014). Probiotic potential and biotherapeutic effects of newly isolated vaginal Lactobacillus acidophilus 36YL strain on cancer cells. Anaerobe..

[2] Riaz Rajoka MS, Shi J, Zhu J, Shao D, Huang Q, Yang H (2017). Capacity of lactic acid bacteria in immunity enhancement and cancer prevention. Appl Microbiol Biotechnol.

[3] Audirac-Chalifour A, Torres-Poveda K, Bahena-Roman M, Tellez-Sosa J, Martinez-Barnetche J, Cortina-Ceballos B (2016). Cervical microbiome and cytokine profile at various stages of cervical Cancer: a pilot study. PLoS One.

[4] Riaz Rajoka MS, Shi J, Mehwish HM, Zhu J, Li Q, Shao D (2017). Interaction between diet composition and gut microbiota and its impact on gastrointestinal tract health. Food Sci Human Wellness.

[5] Kuku S, Fragkos C, McCormack M, Forbes A (2013). Radiation-induced bowel injury: the impact of radiotherapy on survivorship after treatment for gynaecological cancers. Br J Cancer.

[6] Yu H, Xu W, Gong F, Chi B, Chen J, Zhou L (2017). MicroRNA-155 regulates the proliferation, cell cycle, apoptosis and migration of colon cancer cells and targets CBL. Exp Ther Med.

[7] Bray F, Ferlay J, Soerjomataram I, Siegel RL, Torre LA, Jemal A (2018). Global cancer statistics 2018: GLOBOCAN estimates of incidence and mortality worldwide for 36 cancers in 185 countries. CA Cancer J Clin.

[8] Bermudez A, Bhatla N, Leung E (2015). Cancer of the cervix uteri. Int J Gynaecol Obstet.

[9] Emmett M, Gildea C, Nordin A, Hirschowitz L, Poole J (2018). Cervical cancer - does the morphological subtype affect survival rates?. J Obstet Gynaecol.

[10] Small W, Bacon MA, Bajaj A, Chuang LT, Fisher BJ, Harkenrider MM (2017). Cervical cancer: a global health crisis. Cancer..

[11] Cohen PA, Jhingran A, Oaknin A, Denny L (2019). Cervical cancer. Lancet..

[12] Jamilian M, Farhat P, Foroozanfard F, Afshar Ebrahimi F, Aghadavod E, Bahmani F, et al. Comparison of myo-inositol and metformin on clinical, metabolic and genetic parameters in polycystic ovary syndrome: A randomized controlled clinical trial. Clin Endocrinol (Oxf). 2017;87:194–200. 10.1111/cen.13366 28485095

[13] Foroozanfard F, Talebi M, Samimi M, Mehrabi S, Badehnoosh B, Jamilian M, et al. Effect of two different doses of vitamin D supplementation on metabolic profiles of insulin-resistant patients with polycystic ovary syndrome: A randomized, double-blind, placebo-controlled trial. Horm Metab Res. 2017;49:612–7. 10.1055/s-0043-112346 28679142

[14] Pogoda CS, Roden RB, Garcea RL (2016). Immunizing against Anogenital Cancer: HPV vaccines. PLoS Pathog.

[15] Siegel RL, Miller KD, Jemal A (2016). Cancer statistics, 2016. CA Cancer J Clin.

[16] Bosch FX, Qiao Y-L, Castellsagué X (2006). CHAPTER 2 the epidemiology of human papillomavirus infection and its association with cervical cancer. Int J Gynecol Obstet.

[17] Crosbie EJ, Einstein MH, Franceschi S, Kitchener HC (2013). Human papillomavirus and cervical cancer. Lancet..

[18] Furumoto H, Irahara M (2002). Human papilloma virus (HPV) and cervical cancer. J Med Invest.

[19] Clifford GM, Smith JS, Plummer M, Munoz N, Franceschi S (2003). Human papillomavirus types in invasive cervical cancer worldwide: a meta-analysis. Br J Cancer.

[20] Li N, Franceschi S, Howell-Jones R, Snijders PJ, Clifford GM (2011). Human papillomavirus type distribution in 30,848 invasive cervical cancers worldwide: variation by geographical region, histological type and year of publication. Int J Cancer.

[21] Finocchario-Kessler S, Wexler C, Maloba M, Mabachi N, Ndikum-Moffor F, Bukusi E (2016). Cervical cancer prevention and treatment research in Africa: a systematic review from a public health perspective. BMC Womens Health.

[22] Van Kriekinge G, Castellsague X, Cibula D, Demarteau N (2014). Estimation of the potential overall impact of human papillomavirus vaccination on cervical cancer cases and deaths. Vaccine..

[23] Wu ES, Jeronimo J, Feldman S (2017). Barriers and challenges to treatment alternatives for early-stage cervical Cancer in lower-resource settings. J Glob Oncol.

[24] Sankaranarayanan R, Budukh AM, Rajkumar R (2001). Effective screening programmes for cervical cancer in low- and middle-income developing countries. Bull World Health Organ.

[25] WHO Guidelines for Screening and Treatment of Precancerous Lesions for Cervical Cancer Prevention. Geneva: World Health Organization; 2013. WHO Guidelines Approved by the Guidelines Review Committee. 24716265

[26] Denny L, Quinn M, Sankaranarayanan R (2006). Chapter 8: screening for cervical cancer in developing countries. Vaccine.

[27] Mishra GA, Pimple SA, Shastri SS (2011). An overview of prevention and early detection of cervical cancers. Indian J Med Paediatr Oncol.

[28] Lim AW, Ramirez AJ, Hamilton W, Sasieni P, Patnick J, Forbes LJ (2014). Delays in diagnosis of young females with symptomatic cervical cancer in England: an interview-based study. Br J Gen Pract.

[29] Stapley S, Hamilton W (2011). Gynaecological symptoms reported by young women: examining the potential for earlier diagnosis of cervical cancer. Fam Pract.

[30] Alshati A, Almohammedawi M, Sachdev MS, Kachaamy T (2019). Endoscopic management of colovaginal fistulas in advanced cancer patients. VideoGIE.

[31] Sun R, Koubaa I, Limkin EJ, Dumas I, Bentivegna E, Castanon E (2018). Locally advanced cervical cancer with bladder invasion: clinical outcomes and predictive factors for vesicovaginal fistulae. Oncotarget..

[32] Li H, Wu X, Cheng X (2016). Advances in diagnosis and treatment of metastatic cervical cancer. J Gynecol Oncol.

[33] Kumar L, Harish P, Malik PS, Khurana S (2018). Chemotherapy and targeted therapy in the management of cervical cancer. Curr Probl Cancer.

[34] Ranieri G, Patruno R, Ruggieri E, Montemurro S, Valerio P, Ribatti D (2006). Vascular endothelial growth factor (VEGF) as a target of bevacizumab in cancer: from the biology to the clinic. Curr Med Chem.

[35] Ozen M, Dinleyici EC (2015). The history of probiotics: the untold story. Benef Microbes.

[36] Azad MAK, Sarker M, Li T, Yin J (2018). Probiotic species in the modulation of gut microbiota: An overview. Biomed Res Int.

[37] Hill C, Guarner F, Reid G, Gibson GR, Merenstein DJ, Pot B (2014). Expert consensus document. The international scientific Association for Probiotics and Prebiotics consensus statement on the scope and appropriate use of the term probiotic. Nat Rev Gastroenterol Hepatol.

[38] Sanders ME (2015). Probiotics in 2015: their scope and use. J Clin Gastroenterol.

[39] Brown AC, Valiere A (2004). Probiotics and medical nutrition therapy. Nutr Clin Care.

[40] Shanahan F, Dinan TG, Ross P, Hill C (2012). Probiotics in transition. Clin Gastroenterol Hepatol.

[41] Yamashita Y, Takeshita T (2017). The oral microbiome and human health. J Oral Sci.

[42] Fijan S (2014). Microorganisms with claimed probiotic properties: an overview of recent literature. Int J Environ Res Public Health.

[43] Jach M, Los R, Maj M, Malm A (2013). Probiotics-technological and manufacturing aspects. POSTEPY MIKROBIOLOGII.

[44] Kristensen NB, Bryrup T, Allin KH, Nielsen T, Hansen TH, Pedersen O (2016). Alterations in fecal microbiota composition by probiotic supplementation in healthy adults: a systematic review of randomized controlled trials. Genome Med.

[45] Rao SC, Athalye-Jape GK, Deshpande GC, Simmer KN, Patole SK (2016). Probiotic supplementation and late-onset Sepsis in preterm infants: a Meta-analysis. Pediatrics..

[46] Bermudez-Brito M, Plaza-Diaz J, Munoz-Quezada S, Gomez-Llorente C, Gil A (2012). Probiotic mechanisms of action. Ann Nutr Metab.

[47] Bin P, Azad MAK, Liu G, Zhu D, Kim SW, Yin Y (2018). Effects of different levels of methionine on sow health and plasma metabolomics during late gestation. Food Funct.

[48] Kau AL, Ahern PP, Griffin NW, Goodman AL, Gordon JI (2011). Human nutrition, the gut microbiome and the immune system. Nature..

[49] Bahmani F, Tajadadi-Ebrahimi M, Kolahdooz, Mazouchi M, Hadaegh H, Jamal AS, et al. The consumption of synbiotic bread containing Lactobacillus sporogenes and inulin affects nitric oxide and malondialdehyde in patients with type 2 diabetes mellitus: randomized, double-blind, placebo-controlled trial. J Am Coll Nutr. 2016;35(6):506–13. 10.1080/07315724.2015.1032443 26430929

[50] George Kerry R, Patra JK, Gouda S, Park Y, Shin HS, Das G (2018). Benefaction of probiotics for human health: a review. J Food Drug Anal.

[51] Kankaanpää P, Sütas Y, Salminen S, Isolauri E (2003). Homogenates derived from probiotic bacteria provide down-regulatory signals for peripheral blood mononuclear cells. Food Chem.

[52] Bodera P, Chcialowski A (2009). Immunomodulatory effect of probiotic bacteria. Recent Patents Inflamm Allergy Drug Discov.

[53] Cayzeele-Decherf A, Pelerin F, Leuillet S, Douillard B, Housez B, Cazaubiel M (2017). Saccharomyces cerevisiae CNCM I-3856 in irritable bowel syndrome: An individual subject meta-analysis. World J Gastroenterol.

[54] Chang HY, Chen JH, Chang JH, Lin HC, Lin CY, Peng CC (2017). Multiple strains probiotics appear to be the most effective probiotics in the prevention of necrotizing enterocolitis and mortality: An updated meta-analysis. PLoS One.

[55] Ganji-Arjenaki M, Rafieian-Kopaei M (2018). Probiotics are a good choice in remission of inflammatory bowel diseases: a meta analysis and systematic review. J Cell Physiol.

[56] Miller LE, Ouwehand AC, Ibarra A (2017). Effects of probiotic-containing products on stool frequency and intestinal transit in constipated adults: systematic review and meta-analysis of randomized controlled trials. Ann Gastroenterol.

[57] Zhang Y, Li L, Guo C, Mu D, Feng B, Zuo X (2016). Effects of probiotic type, dose and treatment duration on irritable bowel syndrome diagnosed by Rome III criteria: a meta-analysis. BMC Gastroenterol.

[58] Alipour B, Homayouni-Rad A, Vaghef-Mehrabany E, Sharif SK, Vaghef-Mehrabany L, Asghari-Jafarabadi M (2014). Effects of Lactobacillus casei supplementation on disease activity and inflammatory cytokines in rheumatoid arthritis patients: a randomized double-blind clinical trial. Int J Rheum Dis.

[59] Sichetti M, De Marco S, Pagiotti R, Traina G, Pietrella D (2018). Anti-inflammatory effect of multistrain probiotic formulation (L. rhamnosus, B. lactis, and B. longum). Nutrition.

[60] Chen J, Chia N, Kalari KR, Yao JZ, Novotna M, Paz Soldan MM (2016). Multiple sclerosis patients have a distinct gut microbiota compared to healthy controls. Sci Rep.

[61] Esmaeili SA, Mahmoudi M, Momtazi AA, Sahebkar A, Doulabi H, Rastin M (2017). Tolerogenic probiotics: potential immunoregulators in systemic lupus Erythematosus. J Cell Physiol.

[62] Kouchaki E, Tamtaji OR, Salami M, Bahmani F, Daneshvar Kakhaki R, Akbari E (2017). Clinical and metabolic response to probiotic supplementation in patients with multiple sclerosis: a randomized, double-blind, placebo-controlled trial. Clin Nutr.

[63] Di Felice G, Barletta B, Butteroni C, Corinti S, Tinghino R, Colombo P (2008). Use of probiotic bacteria for prevention and therapy of allergic diseases: studies in mouse model of allergic sensitization. J Clin Gastroenterol.

[64] Ettinger G, MacDonald K, Reid G, Burton JP (2014). The influence of the human microbiome and probiotics on cardiovascular health. Gut Microbes.

[65] Nwosu FC, Avershina E, Wilson R, Rudi K (2014). Gut microbiota in HIV infection: implication for disease progression and management. Gastroenterol Res Pract.

[66] Rossi M, Mirbagheri S, Keshavarzian A, Bishehsari F (2018). Nutraceuticals in colorectal cancer: a mechanistic approach. Eur J Pharmacol.

[67] Islam SU (2016). Clinical Uses of Probiotics. Medicine.

[68] Hojsak I, Fabiano V, Pop TL, Goulet O, Zuccotti GV, Cokugras FC (2018). Guidance on the use of probiotics in clinical practice in children with selected clinical conditions and in specific vulnerable groups. Acta Paediatr.

[69] Boyle RJ, Robins-Browne RM, Tang ML (2006). Probiotic use in clinical practice: what are the risks?. Am J Clin Nutr.

[70] Burkhardt O, Köhnlein T, Pletz M, Welte T. Saccharomyces boulardii induced sepsis: successful therapy with voriconazole after treatment failure with fluconazole. Scand J Infect Dis. 2005;37(1):69-72. 10.1080/00365540510026454 15764194

[71] Tsai YL, Lin TL, Chang CJ, Wu TR, Lai WF, Lu CC (2019). Probiotics, prebiotics and amelioration of diseases. J Biomed Sci.

[72] Laniewski P, Ilhan ZE, Herbst-Kralovetz MM (2020). The microbiome and gynaecological cancer development, prevention and therapy. Nat Rev Urol.

[73] Kailasapathy K, Chin J (2000). Survival and therapeutic potential of probiotic organisms with reference to Lactobacillus acidophilus and Bifidobacterium spp. Immunol Cell Biol.

[74] Saber A, Alipour B, Faghfoori Z, Yari KA (2017). Cellular and molecular effects of yeast probiotics on cancer. Crit Rev Microbiol.

[75] Wang Y, Wu Y, Wang Y, Xu H, Mei X, Yu D, et al. Antioxidant properties of probiotic Bacteria. Nutrients. 2017;9. 10.3390/nu9050521. 10.3390/nu9050521 PMC5452251 28534820

[76] Chen CC, Lin WC, Kong MS, Shi HN, Walker WA, Lin CY (2012). Oral inoculation of probiotics Lactobacillus acidophilus NCFM suppresses tumour growth both in segmental orthotopic colon cancer and extra-intestinal tissue. Br J Nutr.

[77] Drago L (2019). Probiotics and Colon Cancer. Microorganisms.

[78] Marchese S, Polo A, Ariano A, Velotto S, Costantini S, Severino L. Aflatoxin B1 and M1: Biological Properties and Their Involvement in Cancer Development. Toxins. 2018;10. 10.3390/toxins10060214. 10.3390/toxins10060214 PMC6024316 29794965

[79] El-Nezami HS, Polychronaki NN, Ma J, Zhu H, Ling W, Salminen EK (2006). Probiotic supplementation reduces a biomarker for increased risk of liver cancer in young men from southern China. Am J Clin Nutr.

[80] Liu ZH, Huang MJ, Zhang XW, Wang L, Huang NQ, Peng H (2013). The effects of perioperative probiotic treatment on serum zonulin concentration and subsequent postoperative infectious complications after colorectal cancer surgery: a double-center and double-blind randomized clinical trial. Am J Clin Nutr.

[81] Kassayova M, Bobrov N, Strojny L, Kiskova T, Mikes J, Demeckova V (2014). Preventive effects of probiotic bacteria Lactobacillus plantarum and dietary fiber in chemically-induced mammary carcinogenesis. Anticancer Res.

[82] Andreyev J, Ross P, Donnellan C, Lennan E, Leonard P, Waters C (2014). Guidance on the management of diarrhoea during cancer chemotherapy. Lancet Oncol.

[83] Tian Y, Li M, Song W, Jiang R, Li YQ (2019). Effects of probiotics on chemotherapy in patients with lung cancer. Oncol Lett.

[84] Badehnoosh B, Karamali M, Zarrati M, Jamilian M, Bahmani F, Tajabadi-Ebrahimi M, et al. The effects of probiotic supplementation on biomarkers of inflammation, oxidative stress and pregnancy outcomes in gestational diabetes. J Matern Fetal Neonatal Med. 2018;31(9):1128–36. 10.1080/14767058.2017.1310193 28326881

[85] Wang KD, Xu DJ, Wang BY, Yan DH, Lv Z, Su JR (2018). Inhibitory effect of vaginal Lactobacillus supernatants on cervical Cancer cells. Probiotics Antimicrob Proteins..

[86] Yim EK, Park JS (2005). The role of HPV E6 and E7 oncoproteins in HPV-associated cervical carcinogenesis. Cancer Res Treat.

[87] Nami Y, Abdullah N, Haghshenas B, Radiah D, Rosli R, Khosroushahi AY (2014). Assessment of probiotic potential and anticancer activity of newly isolated vaginal bacterium Lactobacillus plantarum 5BL. Microbiol Immunol.

[88] Riaz Rajoka MS, Zhao H, Lu Y, Lian Z, Li N, Hussain N (2018). Anticancer potential against cervix cancer (HeLa) cell line of probiotic Lactobacillus casei and Lactobacillus paracasei strains isolated from human breast milk. Food Funct.

[89] Sungur T, Aslim B, Karaaslan C, Aktas B (2017). Impact of exopolysaccharides (EPSs) of Lactobacillus gasseri strains isolated from human vagina on cervical tumor cells (HeLa). Anaerobe..

[90] Liu CT, Chu FJ, Chou CC, Yu RC (2011). Antiproliferative and anticytotoxic effects of cell fractions and exopolysaccharides from Lactobacillus casei 01. Mutat Res.

[91] Nouri Z, Karami F, Neyazi N, Modarressi MH, Karimi R, Khorramizadeh MR (2016). Dual anti-metastatic and anti-proliferative activity assessment of two probiotics on HeLa and HT-29 cell lines. Cell J.

[92] Cha MK, Lee DK, An HM, Lee SW, Shin SH, Kwon JH (2012). Antiviral activity of Bifidobacterium adolescentis SPM1005-a on human papillomavirus type 16. BMC Med.

[93] Li C, Jia L, Yu Y, Jin L (2019). Lactic acid induced microRNA-744 enhances motility of SiHa cervical cancer cells through targeting ARHGAP5. Chem Biol Interact.

[94] Einhorn N, Trope C, Ridderheim M, Boman K, Sorbe B, Cavallin-Stahl E (2003). A systematic overview of radiation therapy effects in cervical cancer (cervix uteri). Acta Oncol.

[95] Kao MS (1995). Intestinal complications of radiotherapy in gynecologic malignancy--clinical presentation and management. Int J Gynaecol Obstet.

[96] Yeoh E, Horowitz M, Russo A, Muecke T, Robb T, Maddox A (1993). Effect of pelvic irradiation on gastrointestinal function: a prospective longitudinal study. Am J Med.

[97] Andreyev J (2005). Gastrointestinal complications of pelvic radiotherapy: are they of any importance?. Gut..

[98] Visich KL, Yeo TP (2010). The prophylactic use of probiotics in the prevention of radiation therapy-induced diarrhea. Clin J Oncol Nurs.

[99] Linn YH, Thu KK, Win NHH (2019). Effect of probiotics for the prevention of acute radiation-induced Diarrhoea among cervical Cancer patients: a randomized double-blind placebo-controlled study. Probiotics Antimicrob Proteins.

[100] Giralt J, Regadera JP, Verges R, Romero J, de la Fuente I, Biete A (2008). Effects of probiotic Lactobacillus casei DN-114 001 in prevention of radiation-induced diarrhea: results from multicenter, randomized, placebo-controlled nutritional trial. Int J Radiat Oncol Biol Phys.

[101] Delia P, Sansotta G, Donato V, Frosina P, Messina G, De Renzis C (2007). Use of probiotics for prevention of radiation-induced diarrhea. World J Gastroenterol.

[102] Kaur IP, Chopra K, Saini A (2002). Probiotics: potential pharmaceutical applications. Eur J Pharm Sci.

[103] Walsham NE, Sherwood RA (2016). Fecal calprotectin in inflammatory bowel disease. Clin Exp Gastroenterol.

[104] De Loera Rodriguez LH, Ortiz GG, Rivero Moragrega P, Velazquez Brizuela IE, Santoscoy Gutierrez JF, Rincon Sanchez AR (2018). Effect of symbiotic supplementation on fecal calprotectin levels and lactic acid bacteria, Bifidobacteria, Escherichia coli and Salmonella DNA in patients with cervical cancer. Nutr Hosp.

[105] Chitapanarux I, Chitapanarux T, Traisathit P, Kudumpee S, Tharavichitkul E, Lorvidhaya V (2010). Randomized controlled trial of live lactobacillus acidophilus plus bifidobacterium bifidum in prophylaxis of diarrhea during radiotherapy in cervical cancer patients. Radiat Oncol.

[106] Zhou Z, Chen X, Sheng H, Shen X, Sun X, Yan Y (2020). Engineering probiotics as living diagnostics and therapeutics for improving human health. Microb Cell Factories.

[107] Danino T, Prindle A, Kwong GA, Skalak M, Li H, Allen K (2015). Programmable probiotics for detection of cancer in urine. Sci Transl Med.

[108] Mao N, Cubillos-Ruiz A, Cameron DE, Collins JJ. Probiotic strains detect and suppress cholera in mice. Sci Transl Med. 2018;10. 10.1126/scitranslmed.aao2586. 10.1126/scitranslmed.aao2586 PMC7821980 29899022

[109] Chew SY, Cheah YK, Seow HF, Sandai D, Than LT (2015). Probiotic Lactobacillus rhamnosus GR-1 and Lactobacillus reuteri RC-14 exhibit strong antifungal effects against vulvovaginal candidiasis-causing Candida glabrata isolates. J Appl Microbiol.

[110] Kohler GA, Assefa S, Reid G (2012). Probiotic interference of Lactobacillus rhamnosus GR-1 and Lactobacillus reuteri RC-14 with the opportunistic fungal pathogen Candida albicans. Infect Dis Obstet Gynecol.

[111] Perisic Z, Perisic N, Golocorbin Kon S, Vesovic D, Jovanovic AM, Mikov M (2011). The influence of probiotics on the cervical malignancy diagnostics quality. Vojnosanit Pregl.

[112] Ou YC, Fu HC, Tseng CW, Wu CH, Tsai CC, Lin H (2019). The influence of probiotics on genital high-risk human papilloma virus clearance and quality of cervical smear: a randomized placebo-controlled trial. BMC Womens Health.

[113] Tsuda N, Watari H, Ushijima K (2016). Chemotherapy and molecular targeting therapy for recurrent cervical cancer. Chin J Cancer Res.

[114] Gui QF, Lu HF, Zhang CX, Xu ZR, Yang YH (2015). Well-balanced commensal microbiota contributes to anti-cancer response in a lung cancer mouse model. Genet Mol Res.

[115] Sivan A, Corrales L, Hubert N, Williams JB, Aquino-Michaels K, Earley ZM (2015). Commensal Bifidobacterium promotes antitumor immunity and facilitates anti-PD-L1 efficacy. Science..

[116] Okawa T, Niibe H, Arai T, Sekiba K, Noda K, Takeuchi S (1993). Effect of LC9018 combined with radiation therapy on carcinoma of the uterine cervix. A phase III, multicenter, randomized, controlled study. Cancer..

[117] Negi D, Singh A, Joshi N, Mishra N. Cisplatin and Probiotic Biomass Loaded Pessaries for the Management of Cervical Cancer. Anticancer Agents Med Chem. 2020;20(5):589–98 10.2174/1871520619666191211110640. 10.2174/1871520619666191211110640 31823703

[118] Kim SN, Lee WM, Park KS, Kim JB, Han DJ, Bae J (2015). The effect of Lactobacillus casei extract on cervical cancer cell lines. Contemp Oncol (Pozn).

[119] Villeger R, Lopes A, Carrier G, Veziant J, Billard E, Barnich N, et al. Intestinal microbiota: a novel target to improve anti-tumor treatment? Int J Mol Sci. 2019;20. 10.3390/ijms20184584. 10.3390/ijms20184584 PMC6770123 31533218

[120] Pardini B, De Maria D, Francavilla A, Di Gaetano C, Ronco G, Naccarati A (2018). MicroRNAs as markers of progression in cervical cancer: a systematic review. BMC Cancer.

[121] Rahbar Saadat Y, Pourseif MM, Zununi Vahed S, Barzegari A, Omidi Y, Barar J. Modulatory role of vaginal-isolated Lactococcus lactis on the expression of miR-21, miR-200b, and TLR-4 in CAOV-4 cells and in Silico revalidation. Probiotics Antimicrob Proteins. 2019. 10.1007/s12602-019-09596-9. 10.1007/s12602-019-09596-9 31797280

[122] Rodriguez-Nogales A, Algieri F, Garrido-Mesa J, Vezza T, Utrilla MP, Chueca N (2018). The Administration of Escherichia coli Nissle 1917 ameliorates development of DSS-induced colitis in mice. Front Pharmacol.

[123] Kalani M, Hodjati H, Sajedi Khanian M, Doroudchi M (2016). Lactobacillus acidophilus increases the anti-apoptotic micro RNA-21 and decreases the pro-inflammatory micro RNA-155 in the LPS-treated human endothelial cells. Probiotics Antimicrob Proteins..

[124] Tilborghs S, Corthouts J, Verhoeven Y, Arias D, Rolfo C, Trinh XB (2017). The role of nuclear factor-kappa B signaling in human cervical cancer. Crit Rev Oncol Hematol.

[125] Yousefi B, Eslami M, Ghasemian A, Kokhaei P, Salek Farrokhi A, Darabi N (2019). Probiotics importance and their immunomodulatory properties. J Cell Physiol.

[126] Kim DE, Kim JK, Han SK, Jang SE, Han MJ, Kim DH (2019). Lactobacillus plantarum NK3 and Bifidobacterium longum NK49 alleviate bacterial Vaginosis and osteoporosis in mice by suppressing NF-kappaB-linked TNF-alpha expression. J Med Food.

[127] Lee HJ, Lim SM, Kim DH (2018). Lactobacillus johnsonii CJLJ103 attenuates scopolamine-induced memory impairment in mice by increasing BDNF expression and inhibiting NF-kappaB activation. J Microbiol Biotechnol.

[128] Kim WG, Kim HI, Kwon EK, Han MJ, Kim DH (2018). Lactobacillus plantarum LC27 and Bifidobacterium longum LC67 mitigate alcoholic steatosis in mice by inhibiting LPS-mediated NF-kappaB activation through restoration of the disturbed gut microbiota. Food Funct.

[129] Hegazy SK, El-Bedewy MM (2010). Effect of probiotics on pro-inflammatory cytokines and NF-kappaB activation in ulcerative colitis. World J Gastroenterol.

[130] Yang SF, Yuan SS, Yeh YT, Wu MT, Su JH, Hung SC (2005). The role of p-STAT3 (ser727) revealed by its association with Ki-67 in cervical intraepithelial neoplasia. Gynecol Oncol.

[131] Chen CL, Hsieh FC, Lieblein JC, Brown J, Chan C, Wallace JA (2007). Stat3 activation in human endometrial and cervical cancers. Br J Cancer.

[132] Shukla S, Shishodia G, Mahata S, Hedau S, Pandey A, Bhambhani S (2010). Aberrant expression and constitutive activation of STAT3 in cervical carcinogenesis: implications in high-risk human papillomavirus infection. Mol Cancer.

[133] Morgan EL, Macdonald A (2019). Autocrine STAT3 activation in HPV positive cervical cancer through a virus-driven Rac1-NFkappaB-IL-6 signalling axis. PLoS Pathog.

[134] Do EJ, Hwang SW, Kim SY, Ryu YM, Cho EA, Chung EJ (2016). Suppression of colitis-associated carcinogenesis through modulation of IL-6/STAT3 pathway by balsalazide and VSL#3. J Gastroenterol Hepatol.

[135] Zhou X, Qi W, Hong T, Xiong T, Gong D, Xie M (2018). Exopolysaccharides from Lactobacillus plantarum NCU116 regulate intestinal barrier function via STAT3 signaling pathway. J Agric Food Chem.

[136] Hou Q, Ye L, Liu H, Huang L, Yang Q, Turner JR (2018). Lactobacillus accelerates ISCs regeneration to protect the integrity of intestinal mucosa through activation of STAT3 signaling pathway induced by LPLs secretion of IL-22. Cell Death Differ.

